# Short-term oral zinc supplementation enhances Natural Killer cell functionality and decreases circulating Innate Lymphoid Cell counts and frequencies in healthy young adults

**DOI:** 10.1186/s12967-025-06259-y

**Published:** 2025-03-14

**Authors:** Lara Amling, Lothar Rink, Sabrina B. Bennstein

**Affiliations:** Institute of Immunology, Medical Faculty, RWTH Aachen University, Aachen, Germany

**Keywords:** Dietary supplements, Trace element, Zinc, ILCs, NK cells, Zinc supplementation, Nutrition, Vegetarians, Vegans, Healthy young adults

## Abstract

**Background:**

Zinc is an essential trace element with high importance for immune function. Previous research has shown that vegetarians and vegans are at increased risk of zinc deficiency, due to low zinc bioavailability in plant-based food. However, its effects on immune parameters in healthy adults following these diets remain largely unexplored. Therefore, this study investigated the effects of dietary patterns, serum zinc levels, and short-term oral zinc supplementation on Natural Killer (NK) cells, circulating Innate Lymphoid Cells (cILCs), and NK cell functionality in omnivores, vegetarians, and vegans.

**Methods:**

A total of 54 study participants, including 21 omnivores, 18 vegetarians, and 15 vegans were enrolled in our study. NK cell and cILC counts and frequencies were analyzed by flow cytometry and NK cell cytotoxicity assay was performed and compared between the three dietary cohorts as well as between zinc adequate (ZA) and zinc deficient (ZD) individuals. Based on serum zinc concentrations and/or Food Frequency Questionnaire (FFQ) scores, study participants classified as ZD were supplemented with 10 mg zinc daily for 14 days. After this period, the same experiments were performed.

**Results:**

Our results show that neither dietary patterns nor baseline zinc levels significantly affect cILC or NK cell counts, frequencies, or NK cell cytotoxicity. However, short-term oral zinc supplementation significantly reduced cILC counts and frequencies, while enhancing NK cell functionality. Here, NK cell cytotoxicity is significantly positively correlated, whereas cILC counts are negatively correlated with serum zinc concentrations. Remarkably, 72% of all study participants, including 48% of omnivores, were classified as ZD.

**Conclusions:**

Since proper NK cell functionality is required for early defense against infected or malignant cells, and cILCs act as progenitors to replenish tissue resident ILCs, which are crucial for tissue homeostasis and barrier integrity, our results suggest that routine zinc supplementation might be a simple yet effective strategy to enhance immune defense and potentially prevent diseases across different dietary groups.

**Trial registration:**

The study was approved and registered by the Institutional Ethics Committee of the Medical Faculty of RWTH Aachen University on the 19th of July 2023 (study numbers: EK 23–148 and EK 23–234, CTC number: 23–163).

**Supplementary Information:**

The online version contains supplementary material available at 10.1186/s12967-025-06259-y.

## Background

### Natural Killer (NK) cells

NK cells are part of the innate immune system described as CD3^−^, CD94^+^ and CD56^+^ cells constituting for 10 to 15% of all lymphocytes [1–3]. Their ability to mediate antigen-independent cytotoxicity makes them essential for the early defense against infected or malignant cells [4]. NK cells exert their effects primarily through the release of cytotoxic granules containing perforin and granzyme, or through ligands such as Fas Ligand (FasL) and Tumor Necrosis Factor-Related Apoptosis-Inducing Ligand (TRAIL), both of which induce apoptosis in target cells [5, 6]. Simultaneously, they secrete cytokines, such as Interferon-γ (IFN-γ) [7], which enhances the immune response by activating macrophages and promoting the adaptive immune system’s response via T cells [8]. NK cell activation is primarily driven by the principle of “missing-self”, involving the recognition of aberrant or missing human leucocyte antigen (HLA) class I molecules, but also results from the integration of multiple activating and inhibitory signals [2, 9]. Together with CD3^+^ T-Lymphocytes and CD19^+^ B-Lymphocytes, NK cells are the predominant immune cells within the lymphoid lineage [10]. However, rare populations of innate immune cells, including Innate Lymphoid Cells (ILCs), have been identified within the last decade.

### Innate Lymphoid Cells (ILCs)

ILCs lack lineage-defining cell surface receptors and express the Interleukin-7 (IL-7) receptor (IL-7R, CD127) [11]. Based on the expression of CD117 and CRTH2, ILCs have been divided into three distinct subsets: ILC1 (CD117^−^ CRTH2^−^), ILC2 (CD117^+^/^−^, CRTH2^+^) and ILC3 (CD117^+^, CRTH2^−^) [12, 13]. Unlike tissue resident ILCs (tILCs), which reflect the functionality of established CD4^+^ T helper cell subsets, the precise functions of human circulating ILCs (cILCs) in peripheral blood are not yet fully understood [14]. Only cILC2s are known to functionally resemble their tissue resident counterparts, while cILC1s and cILC3s remain largely unresponsive in terms of effector function [15–18]. In particular, cILC1s have been described as NK cell progenitors contributing to the generation of KIR^+^NKG2A^−^ NK cells [18], whereas cILC3s have a broad differentiation potential towards all three cILC subsets as well as NK cells [15]. Reported cILC frequencies range from 0.1 to 1% of lymphocytes, with total cILC counts ranging from 2 to 45 cells per µl blood. However, to date, only a few studies have focused on factors influencing cILC counts and frequencies [14].

### Zinc deficiency

Zinc is an essential trace element with a broad range of functions within the immune system [19]. Disruptions in zinc homeostasis, whether due to insufficient dietary uptake or malabsorption, can lead to zinc deficiency (ZD) [20], known to impair various immune responses. For example, mild zinc deficiency could increase susceptibility to infections such as COVID-19 [21, 22] or might contribute to the development of allergic diseases [23]. To assess one’s individual zinc status and the risk of zinc deficiency, two methods are commonly used in the literature and similar studies [24]. Serum zinc concentration, measured by atomic absorption spectroscopy (AAS), is the most widely used marker for evaluating human zinc status [24] and is considered to best reflect physiological zinc conditions. Serum zinc concentrations below 70 µg/dl are considered ZD [24]. Additionally, dietary zinc uptake can be quantified using a zinc-specific 18-item Food Frequency Questionnaire (FFQ), for example within the Zinc-App [25], which calculates a phytate-corrected Adjusted Zinc Diet Score (AZDS) [26]. Here, a AZDS below 113 points is considered ZD. Individuals eligible for zinc supplementation are defined as those with an AZDS score below 113 points and/or serum zinc concentrations less than 70 µg/dl.

### Zinc’s role in innate immune function

So far little is known on the effects of zinc on NK cells and ILCs. Given zinc’s important role in immune homeostasis, we hypothesize that zinc may influence cILCs, which are progenitors for other immune cells, including NK cells [14]. This suggests that their regulation could impact broader immune functions. A recent in vitro study from our institute demonstrated that NK cell cytotoxicity could be significantly enhanced following short (1 h) zinc supplementation of Peripheral Blood Mononuclear Cells (PBMCs) [27].

Zinc deficiency is common not only among the elderly [24], but also in young, healthy individuals who follow a vegetarian or vegan diet. These dietary patterns are often associated with lower dietary zinc uptake, while omnivorous diets are more likely to ensure an adequate zinc status [26, 28]. To this date, only limited research exists on how zinc deficiency or supplementation affects immune functions in vegetarians or vegans. So far, no in vivo study has investigated NK cell functionality, cILC and NK cell counts and frequencies in young, healthy individuals following an omnivorous, vegetarian, or vegan diet. Therefore, the aim of this study was to investigate potential differences in cILC and NK cell counts and frequencies as well as NK cell functionality based on dietary habits and the initial zinc status of the study participants. In addition, we sought to evaluate the effects of oral zinc supplementation for 14 days on these parameters in study participants classified as ZD based on FFQ scores and/or serum zinc measurements. To address these questions, we analyzed primary human PBMCs from young and healthy volunteers following either an omnivorous, vegetarian, or vegan diet for at least three months. Study participants meeting the criteria for zinc deficiency were daily supplemented with 50 mg of zinc-aspartate, corresponding to 10 mg of available zinc. Specifically, the 10 mg/day dose was selected as it aligns with prior and similar studies assessing zinc’s physiological effects [24] and falls within the recommended dietary allowance range for adults [20], ensuring safety and efficacy in supplementation.

## Materials and methods

### Recruitment and study design

**Fig. 1:**
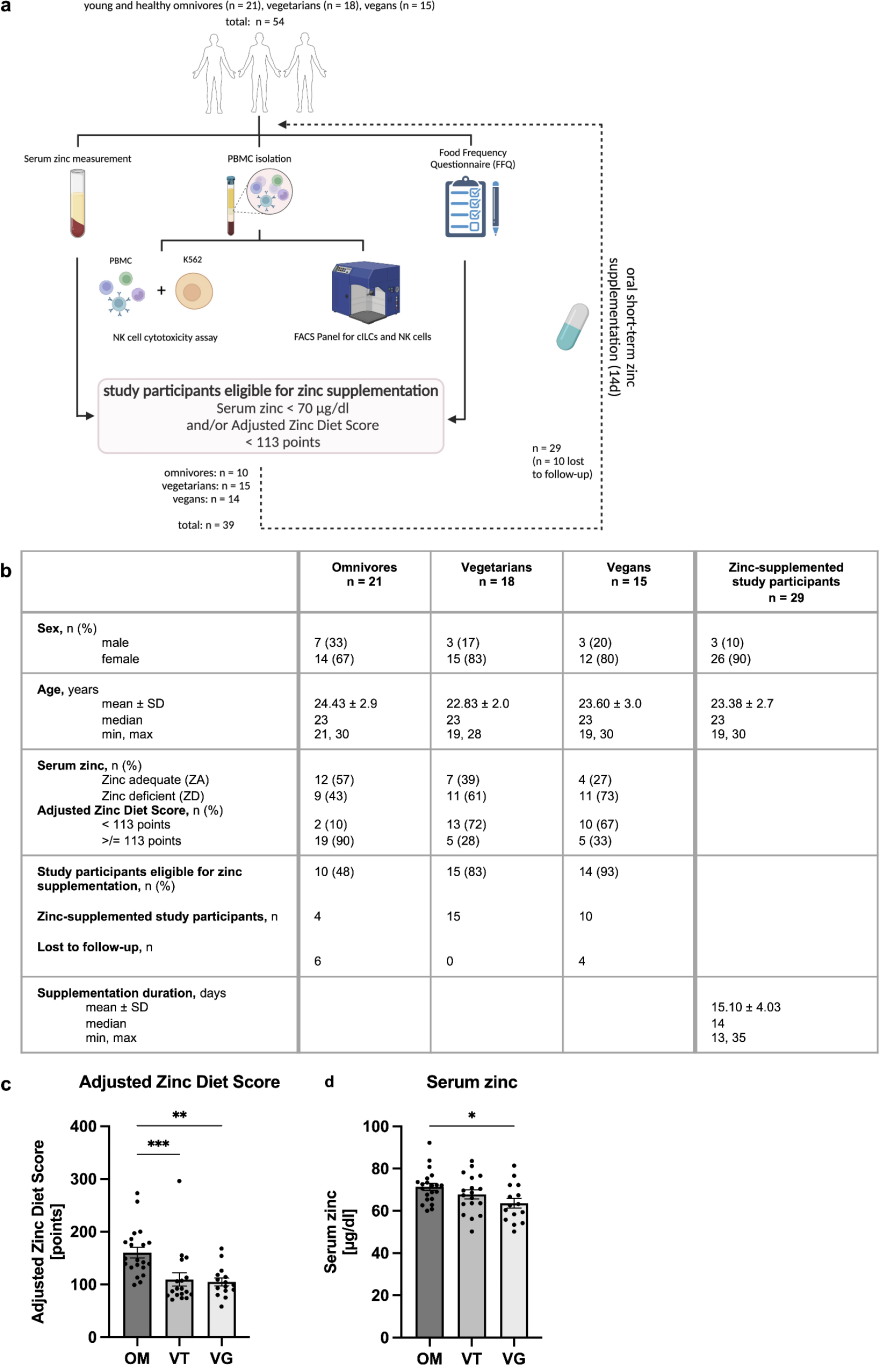
Study design flow chart and characterization of the study cohort. PBMCs were isolated by density gradient from peripheral blood of young and healthy omnivores (*n* = 21), vegetarians (*n* = 18), and vegans (*n* = 15). NK cell and cILC counts and frequencies were analyzed by flow cytometry, and NK cell cytotoxicity assay was performed. Zinc status of all study participants was assessed by measuring serum zinc concentrations and using a zinc-specific Food Frequency Questionnaire (FFQ) to estimate net dietary zinc uptake by calculating a phytate-corrected Adjusted Zinc Diet Score (AZDS). A total of 29 study participants that were previously classified as ZD based on serum zinc concentrations and/or FFQ scores received short-term oral zinc supplementation for 14 days. After this period, the same experiments were performed **(A)**. Characteristics of the study cohort are shown **(B)**. Bar graphs show the omnivorous (OM) (*n* = 21), vegetarian (VT) (*n* = 18), and vegan (VG) (*n* = 15) study cohort in terms of net dietary zinc uptake measured by the AZDS **(C)** and serum zinc concentrations **(D)**. All data are considered as unpaired parameters and are presented as the mean ± SEM **(C and D)**. Statistical significance was calculated by Kruskal-Wallis test followed by Dunn’s multiple comparison test in case of non-normal distribution **(C)** and ordinary one-way ANOVA followed by Tukey’s multiple comparison test in case of normal distribution **(D)**. * *p*-value < 0.05, ** *p*-value < 0.01, *** *p*-value < 0.001

Young and healthy omnivorous, vegetarian, or vegan study participants aged 19 to 30 years were recruited between May and August 2024 (Fig. 1A). The study was reviewed and approved by the Institutional Ethics Committee of the Medical Faculty of RWTH Aachen University (EK 23–148 and EK 23–234). The experimental setup is shown in Fig. 1A. The diet had to be followed at least for the last three months before initial blood draw. As shown in similar studies [24], net dietary zinc uptake was assessed using a well-established zinc-specific 18-item Food Frequency Questionnaire (FFQ) within the Zinc-App [25], which analyzes the frequency, portion size, and quantity of specific foods. Based on this, the questionnaire calculates a phytate-corrected Adjusted Zinc Diet Score [26]. As previously described, zinc deficiency was further assessed by serum zinc measurement [24]. Study participants eligible for short-term oral zinc supplementation had either a serum zinc concentration < 70 µg/dl and/or an Adjusted Zinc Diet Score < 113 points [24].

Study participants meeting these criteria were daily supplemented with 50 mg of zinc-aspartate, corresponding to 10 mg of available zinc (Unizink, Köhler Pharma GmbH), for 14 days [24]. After this period, the same experiments were performed. The characterization of the study cohort is shown in Fig. 1B.

### Serum zinc measurement

For serum zinc measurement, blood was collected in 9 ml serum monovettes (Sarstedt) and was centrifuged at 1841xG for 10 min. Subsequently, 1 ml of serum was diluted equally with deionized water in Eppendorf tubes. Serum zinc concentrations were determined by flame atomic absorption spectrometry (AAS) using an AAnalyst 800 (Perkin-Elmer) as previously described [29].

### Isolation of peripheral blood mononuclear cells (PBMC)

In the afternoon, venous blood was drawn from the study participants and heparinized with 50 U heparin (Braun) per ml blood. The blood was left at room temperature overnight. The next morning, it was diluted equally with Dulbecco’s phosphate-buffered saline (1x PBS, Sigma/Aldrich). After the isolation of PBMCs using Ficoll-density-gradient-centrifugation (Lymphocyte-Separation Medium, Density: 1.077 g/ml, Capricorn Scientific), erythrocytes were lysed with 5 ml of erythrocytes-lysing-buffer containing 1.5 M ammonium chloride, 100 mM sodium hydrogen carbonate and 10 mM triplex-111. PBMCs were washed thrice with 1x PBS.

### NK cell cytotoxicity assay

After PBMC isolation, NK cell assay was performed as previously described [27, 30]. Briefly, 2.5 × 10^6^ PBMCs were mixed with 2 × 10^5^ carboxyfluorescein diacetate succinimidyl ester (CFDA-SE) (Thermo Fisher Scientific) labeled K562 cells (HLA class I deficient erythroleukemic cell line) (effector: target cell ratio: 12.5:1) in RPMI 1640 containing 10% heat-inactivated fetal calf serum (FCS) (Capricon Scientific) and 1% L-Glutamine (Sigma/Aldrich), either stimulated with or without 200 U of Interleukin-2 (IL-2) (Proleukin S, Novartis). 2 × 10^5^ CFDA-SE-labeled K562 cells alone were used as a negative control for spontaneous lysis. The samples were centrifuged at 120 G for 3 min. After 5 h incubation at 37 °C in a 5% humidified CO_2_ atmosphere, K562 cells were stained with 2.5% propidium iodide (PI, Sigma/Aldrich) for 10 min and were measured by flow cytometry using a FACS Calibur (BD Biosciences) to detect dead cells. To analyze the data, we gated on CFDA- and PI-positive K562 cells using FlowJo version 10.10.0. Subsequently, we subtracted the value of PI-positive K562 without PBMCs from the values of K562 cells with PBMCs to determine the target-specific killing rate.

### Flow cytometry analyses of cILCs and NK cells

Freshly isolated PBMCs were adjusted to a final concentration of 2.5 × 10^6^ cells. The antibody mixture containing FITC-conjugated lineage-specific (lin-) and cILC- and NK cell-specific antibodies was added per sample and staining was performed as previously established [13, 31]. The following FITC-conjugated antibody reagents were used to exclude lineage-positive cells: anti-FcεR1α (AER-37 (CRA1)), anti-TCRγδ (B1), anti-CD1a (HI149), anti-TCRαβ (IP26), anti-CD34 (581), anti-CD19 (HIB19), anti-CD14 (HCD14), anti-CD3 (UCHT1), anti-123 (6H6), all from BioLegend. To distinguish NK cell and cILC subsets, we used anti-CD94-PE/Cy7 (DX22), anti-CD56-PE (NCAM), anti-CD117-BV421™ (104D2) and anti-CRTH2-APC/Cy7 (BM16), all from BioLegend. In addition, we used anti-CD127-PE-Cy5 (R34.34) from Beckman Coulter and anti-CD69-APC (FN50) from BD Biosciences. For the first 17 samples, the following antibodies differed from the antibody mixture described above: anti-CD3-FITC (SK7), anti-CD19-FITC (4G7), anti-CD14-FITC (MOP9), anti-CD34-FITC (8G12) and anti-CD56-PE (NKAM16.2), all from BD Biosciences. We excluded the first 17 samples from the NK cell subset analysis regarding the distinction between CD56^dim^ and CD56^bright^ NK cells due to variability observed with the anti-CD56 antibody. No difference was observed for the FITC-coupled antibodies. Subsequent analyses, including the last figure, confirmed that trends and results were consistent regardless of this exclusion, ruling out batch effects. Dilution factors were taken from the previously published protocol [13]. Samples were measured using a FACS Canto II (BD Biosciences) and analyzed using FlowJo version 10.10.0.

For perforin and granzyme B analysis, cryopreserved PBMCs were extracellularly stained with anti-CD3-FITC (UCHT1) and anti-CD94-PE/Cy7 (DX22). Fixation and permeabilization buffer (BioLegend) were added according to the manufacturer’s protocol with anti-Perforin-PE (δG9, eBioscience) or anti-Granzyme B-PE (GB11, eBioscience). Measurements were performed using a Canto II (BD Biosciences) and samples were analyzed using FlowJo version 10.10.0. The gating strategy included gating for CD3^−^CD94^+^ NK cells and adding the geometric mean of perforin and granzyme B to the statistics.

### Calculation of absolute cell counts

3.7 ml of blood was collected in EDTA K3E monovettes (Sarstedt) and lymphocyte cell count was measured using a Sysmex XN-550 analyzer. Based on the lymphocyte cell count per µl blood and total lymphocyte count derived from Canto II, the following formula was used to calculate the absolute cell count of each subset, as in similar studies [31]:

$$\begin{array}{l}\:\frac{\#\:\text{o}\text{f}\:\text{c}\text{e}\text{l}\text{l}\text{s}\:\left(\text{C}\text{a}\text{n}\text{t}\text{o}\:\text{I}\text{I}\right)}{\#\:\text{o}\text{f}\:\text{L}\text{y}\text{m}\text{p}\text{h}\text{o}\text{c}\text{y}\text{t}\text{e}\text{s}\:\left(\text{C}\text{a}\text{n}\text{t}\text{o}\:\text{I}\text{I}\right)}\\*\:Lymphocyte\:cell\:count\:{[x10}^{3}\:pro\:\mu\:l]\:\left(Sysmex\right)\end{array}$$

### Gating strategy

Our gating strategy for human NK cells and cILCs is similar to our previous studies [13, 17, 31]. For the NK cells, we gated on Lin^−^CD94^+^ cells and further subdivided them into CD56^dim^ and CD56^bright^ NK cells based on their CD56 expression. Based on CRTH2 and CD117 expression, Lin^−^CD94^−^CD127^+^ total cILCs were divided into CD117^−^ CRTH2^−^ cILC1, CD117^−/+^CRTH2^+^ cILC2, and CD117^+^CRTH2^−^ cILC3 [13, 14]. We also analyzed CD69 expression on CD56^dim^ and CD56^bright^ NK cells and all cILC subsets.

### IFN-γ-ELISA

After PBMCs were co-cultured with K562 cells for 5 h at 37 °C in a 5% humidified CO_2_ atmosphere to determine NK cell killing, supernatants of PBMCs were harvested and stored in Eppendorf tubes at -80 °C. After all samples were equally diluted with Assay Diluent (BD Biosciences), IFN-γ concentrations of PBMCs before and after zinc supplementation were quantified by OptEIA ELISA (BD Biosciences) according to the manufacturer’s protocol. Absorbance was measured using a Spark 10 M well plate reader (Tecan).

### Power analysis

To calculate the required n-number for our study, we performed a power analysis based on a similar study from our institute investigating the effects of zinc supplementation on CREMα-mediated IL-2 suppression in the elderly. In this study, 10 elderly participants (out of an initial 31 elderly) were supplemented with zinc [24]. Using data from a paired t-test comparing serum zinc concentrations before and after supplementation [24], we calculated a standardized effect size of 1.23 (from a pooled standard deviation = 10.77 and a mean difference = 13.25). The power analysis (effect size: 1.23, significance level: 0.05, target power: 0.8) indicated a minimum sample size of 8 participants per group (16 total). Our study included 54 participants, exceeding this minimum, to allow for initial comparisons between our three study groups (omnivores, vegetarians, and vegans, *n* > 8 per group) before zinc supplementation. We also accounted for participants who might not be eligible for zinc supplementation and potential dropouts to ensure sufficient sample sizes for experiments involving zinc supplementation.

### Statistical analyses

All data was tested for normal distribution using Shapiro-Wilk and Kolmogorov-Smirnov tests. Statistical significances were calculated using GraphPad Prism software (version 10.3.0). The tests used for statistical significances are indicated in the corresponding figure legends. The three study groups (omnivores, vegetarians, and vegans) and the ZA and ZD study participants were considered unpaired parameters, whereas comparisons of data before and after zinc supplementation were considered paired. Correlations were determined by calculating Spearman’s correlation coefficient r and R^2^. Significance is indicated as follows: *p < 0.05 **p < 0.01 ***p < 0.001.

## Results

### Baseline characteristics and initial zinc status of the study cohorts

Given that zinc status is closely related to dietary habits [26, 28], we aimed to investigate the influence of zinc on cell counts and frequencies of cILCs and NK cells, as well as NK cell functionality, among omnivorous, vegetarian, and vegan study participants. Between May and August 2024, a total of 54 study participants, including 21 omnivores, 18 vegetarians, and 15 vegans, mostly students aged 19 to 30 years, were enrolled in the study at RWTH Aachen University Hospital (Fig. 1A). Figure 1B provides detailed information on the age, sex, and initial zinc status of all study participants. Notably, the study cohort independent of their diet was predominantly female (76%).

Analysis of the AZDS revealed that vegetarians (110 points ± 54 (mean ± SD)) and vegans (105 points ± 29) had significantly lower dietary zinc uptake compared to omnivores (161 points ± 46) (Fig. 1C). This finding was further supported by serum zinc measurements, which revealed statistically significant lower serum zinc concentrations in vegans (63.60 µg/dl ± 9.048 (mean ± SD)) compared to omnivores (71.33 µg/dl ± 7.818), while vegetarians showed a non-significant trend towards lower serum zinc concentrations (67.80 µg/dl ± 9.073) (Fig. 1D). Overall, 72% of all study participants were classified as ZD based on their FFQ score and/or serum zinc measurements (48% of omnivores, 83% of vegetarians, and 93% of vegans). In summary, all three study cohorts, including the omnivorous cohort, contained both ZA and ZD individuals.

### No influence of dietary habits and initial zinc status on cILC counts and frequencies

**Fig. 2:**
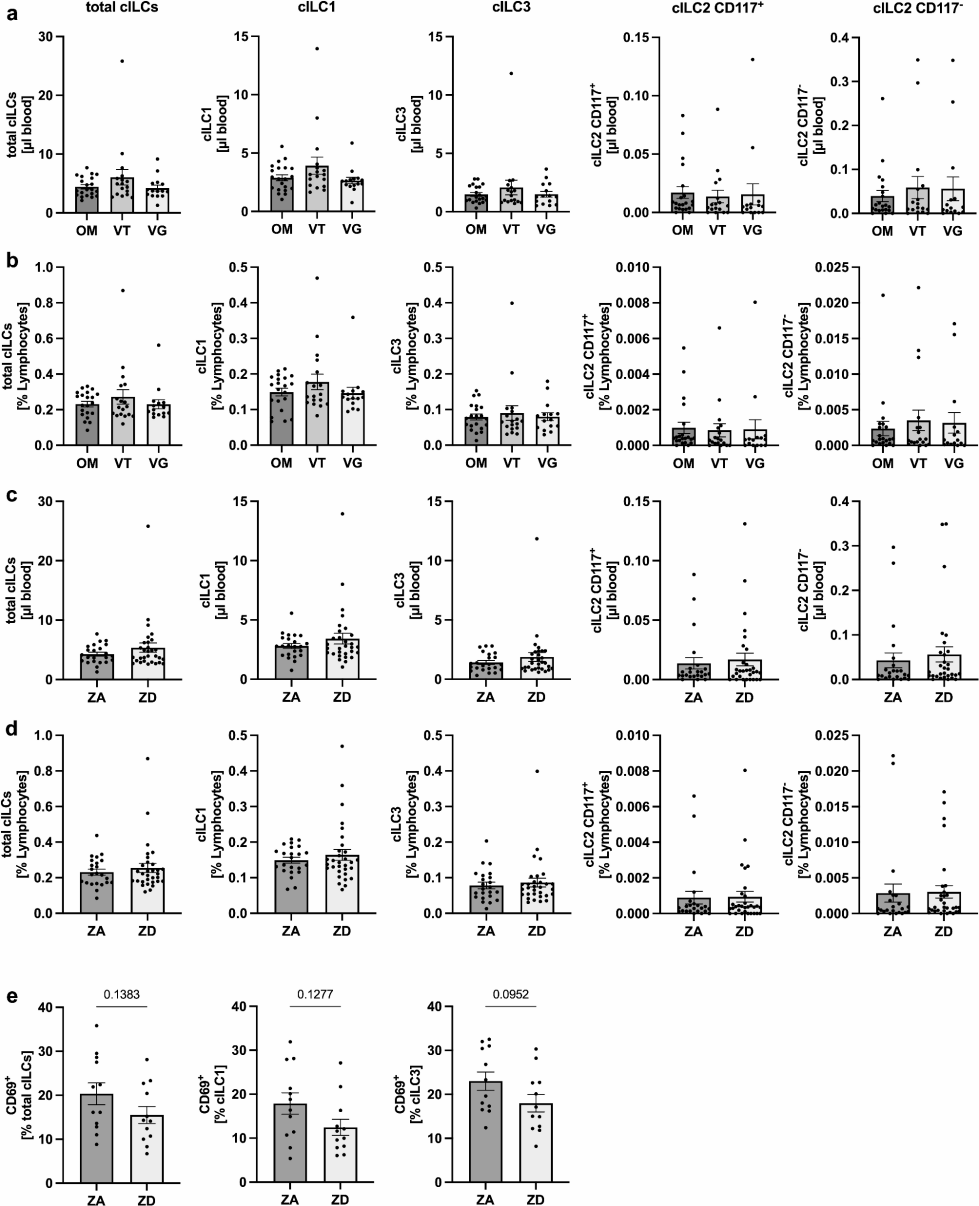
No influence of dietary habits and initial zinc status on cILC counts and frequencies. After isolation of PBMCs by density centrifugation, cILC counts and frequencies were analyzed by flow cytometry. Bar graphs show total cILC, cILC1, cILC2 CD117^+/−^, and cILC3 counts **(A and C)** and frequencies **(B and D)** in omnivorous (OM) (*n* = 21), vegetarian (VT) (*n* = 17–18), and vegan (VG) (*n* = 15) study participants **(A and B)** and between serum zinc adequate (ZA) (*n* = 23) and serum zinc deficient (ZD) (*n* = 30–31) study participants **(C and D)**. Percentages of CD69^+^ total cILCs, CD69^+^ ILC1s, and CD69^+^ ILC3s are compared between ZA (*n* = 12) and ZD (*n* = 12) study participants **(E)**. All data are considered as unpaired parameters and are presented as the mean ± SEM **(A-E)**. Statistical significance was calculated by Kruskal-Wallis test followed by Dunn’s multiple comparison test **(A and B)**, by Mann-Whitney test in case of non-normal distribution **(C-E)** and by unpaired t-test in case of normal distribution **(E**,** left and right graph)**. * *p*-value < 0.05, ** *p*-value < 0.01, *** *p*-value < 0.001

Given that the influence of dietary habits and the corresponding zinc status on cILC counts and frequencies is yet fully unknown, we analyzed PBMCs of the three study cohorts by flow cytometry as described in previous studies [13, 31]. First, we investigated whether cILC counts and fraction of lymphocytes varied between dietary habits. No significant differences were observed between omnivorous, vegetarian, and vegan study participants for total cILC counts (Fig. 2A) or frequencies (Fig. 2B), nor in any of the four cILC subsets (cILC1, cILC2 CD117^+/−^ and cILC3). Next, we sought to assess the impact of initial serum zinc status on cILC counts and frequencies. For this, we initially considered using the three study cohorts and using omnivores as controls with presumably higher serum zinc concentrations. However, as shown in Fig. 1B, zinc status was highly heterogeneous within our three study cohorts. As a result, the omnivores did not serve as an ideal control group, as nearly only half of them were considered ZA. To address this issue, we divided all 54 study participants into two groups based on serum zinc concentrations: Zinc adequate (ZA, serum zinc >/= 70 µg/dl; 76.3 µg/dl ± 5.445 (mean ± SD)) and zinc deficient (ZD, serum zinc < 70 µg/dl; 61.9 µg/dl ± 5.493) study participants. When comparing these two groups, no significant differences in cILC counts (Fig. 2C) and frequencies (Fig. 2D) were found across all cILC subsets. Of note, no sex-specific differences in total cILC counts and frequencies were found between male and female study participants (Supplementary Fig. 1).

To investigate whether there is a difference in the activation of cILCs between ZA and ZD individuals, we further evaluated CD69 expression, a well-established early activation marker and indicator of tissue residency [32]. The results show a non-significant trend in which ZD individuals exhibit lower CD69 expression on total cILCs, cILC1s and cILC3s compared to ZA individuals (Fig. 2E). In conclusion, these findings suggest that neither dietary patterns nor baseline zinc status significantly affect cell counts and frequencies of cILCs in the blood of young and healthy adults while analysis of cILC activation, as assessed by CD69 expression, revealed that zinc deficiency might be associated with reduced cILC activation.

### No influence of dietary habits and initial zinc status on NK cell counts and frequencies

**Fig. 3:**
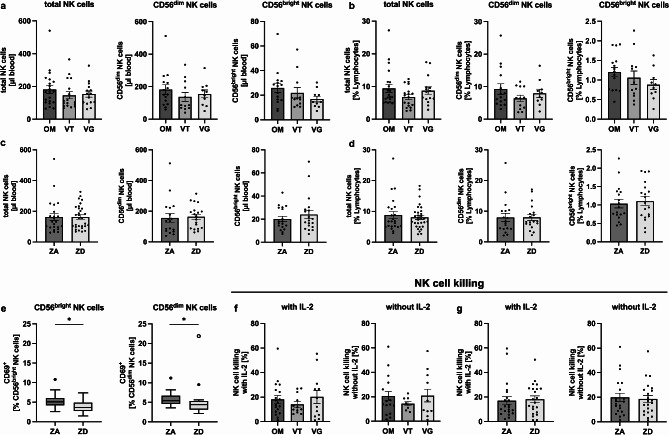
No influence of dietary habits and initial zinc status on NK cell counts, frequencies, and cytotoxicity. After PBMC isolation by density centrifugation, NK cell counts, and frequencies were analyzed by flow cytometry. Bar graphs show total NK cell, CD56^dim^, and CD56^bright^ NK cell counts **(A and C)** and frequencies **(B and D)** in omnivorous (*n* = 15–21), vegetarian (*n* = 12–18), and vegan (*n* = 10–15) study participants **(A and B)** and between serum zinc adequate (ZA) (*n* = 18–23) and serum zinc deficient (ZD) (*n* = 19–31) study participants **(C and D)**. Percentages of CD69^+^ CD56^dim^ and CD56^bright^ NK cells are compared between ZA (*n* = 12) and ZD (*n* = 12) study participants **(E)**. 2.5 × 10^6^ PBMCs and were incubated with CFDA-SE labeled K562 cells with and without IL-2 stimulation and killing of K562 cells was measured after 5 h by flow cytometry using propidium iodide (PI) staining. Bar graphs show the percentages of PI-positive K562 cells, quantifying NK cell killing, between omnivores (*n* = 19), vegetarians (*n* = 11–12), and vegans (*n* = 12) **(F)** and between serum zinc adequate (ZA) (*n* = 20–21) and serum zinc deficient (ZD) (*n* = 22) study participants **(G)**. All data are considered as unpaired parameters and are presented as the mean ± SEM **(A-G)**. Statistical significance was calculated by Kruskal-Wallis test followed by Dunn’s multiple comparison test **(A**,** B**,** and F)** in case on non-normal distribution, by ordinary one-way ANOVA followed by Tukey’s multiple comparison test in case of normal distribution **(B**,** right graph)**, by Mann-Whitney test in case of non-normal distribution **(C-E and G)**, and by unpaired t-test in case of normal distribution **(D**,** right graph and E**,** right graph)**. Significances were calculated with one outlier removed. However, the graph is shown with the outlier **(E)**. * *p*-value < 0.05, ** *p*-value < 0.01, *** *p*-value < 0.001

Based on prior findings that chronic zinc deficiency disrupts T cell lymphopoiesis and erythropoiesis in mice [33], we hypothesized that zinc deficiency could similarly have an impact on human NK cell counts. Therefore, we analyzed NK cell counts and frequencies between omnivores, vegetarians and vegans using flow cytometry. Consistent with the findings for cILC counts and frequencies, there were no significant differences in total NK cell counts (Fig. 3A) and frequencies (Fig. 3B) between the three study cohorts. NK cells were further subdivided into CD56^dim^, and CD56^bright^ NK cell subsets based on CD56 expression, but no differences regarding dietary habits were observed in these subsets either (Fig. 3A/3B). To better assess the potential effect of initial zinc status on NK cell counts and frequencies, participants were again stratified into a ZA and ZD group as described above. No differences in total NK cells or CD56^dim^ and CD56^bright^ NK cell subsets were found between ZA and ZD participants (Fig. 3C/3D) and between males and females (Supplementary Fig. 1). Analysis of CD56^dim^ and CD56^bright^ NK cell activation, as assessed by CD69 expression, revealed a significantly lower percentage of CD69^+^ CD56^dim^ and CD56^bright^ NK cells in individuals classified as ZD compared to ZA (Fig. 3E). In summary, these results indicate that neither dietary habits nor baseline zinc status of our cohort has a significant impact on NK cell counts and frequencies. However, zinc deficiency may be associated with reduced activation of CD56^dim^ and CD56^bright^ NK cells, as assessed by the expression of CD69.

### No influence of dietary habits and initial zinc status on NK cell cytotoxicity

Since dietary habits and baseline zinc status do not influence NK cell counts or frequencies, we next aimed to investigate whether NK cell functionality is affected by a vegetarian or vegan diet and its associated zinc status. A recent in vitro study from our institute demonstrated that NK cell cytotoxicity is regulated in a zinc-dependent manner [27], leading us to hypothesize that individuals following a vegetarian or vegan diet may exhibit a reduced NK cell killing activity. To test this hypothesis, we performed the NK cell cytotoxicity assay following protocols described in previous studies using the HLA class I deficient cell line K562 [18, 27]. Contrary to this hypothesis, our results showed no significant differences in NK cell cytotoxicity, with or without IL-2 stimulation, between omnivores, vegetarians, and vegans (Fig. 3F). Similarly, no significant differences were observed when comparing NK cell cytotoxicity between ZA and ZD participants (Fig. 3G). Of note, NK cell cytotoxicity with and without IL-2 stimulation was higher in males compared to females, with the difference approaching statistical significance (Supplementary Fig. 1). In summary, these results indicate that neither dietary habits nor baseline zinc status of our cohort has a significant impact on NK cell cytotoxicity.

### Short-term oral zinc supplementation enhances NK cell cytotoxicity

**Fig. 4:**
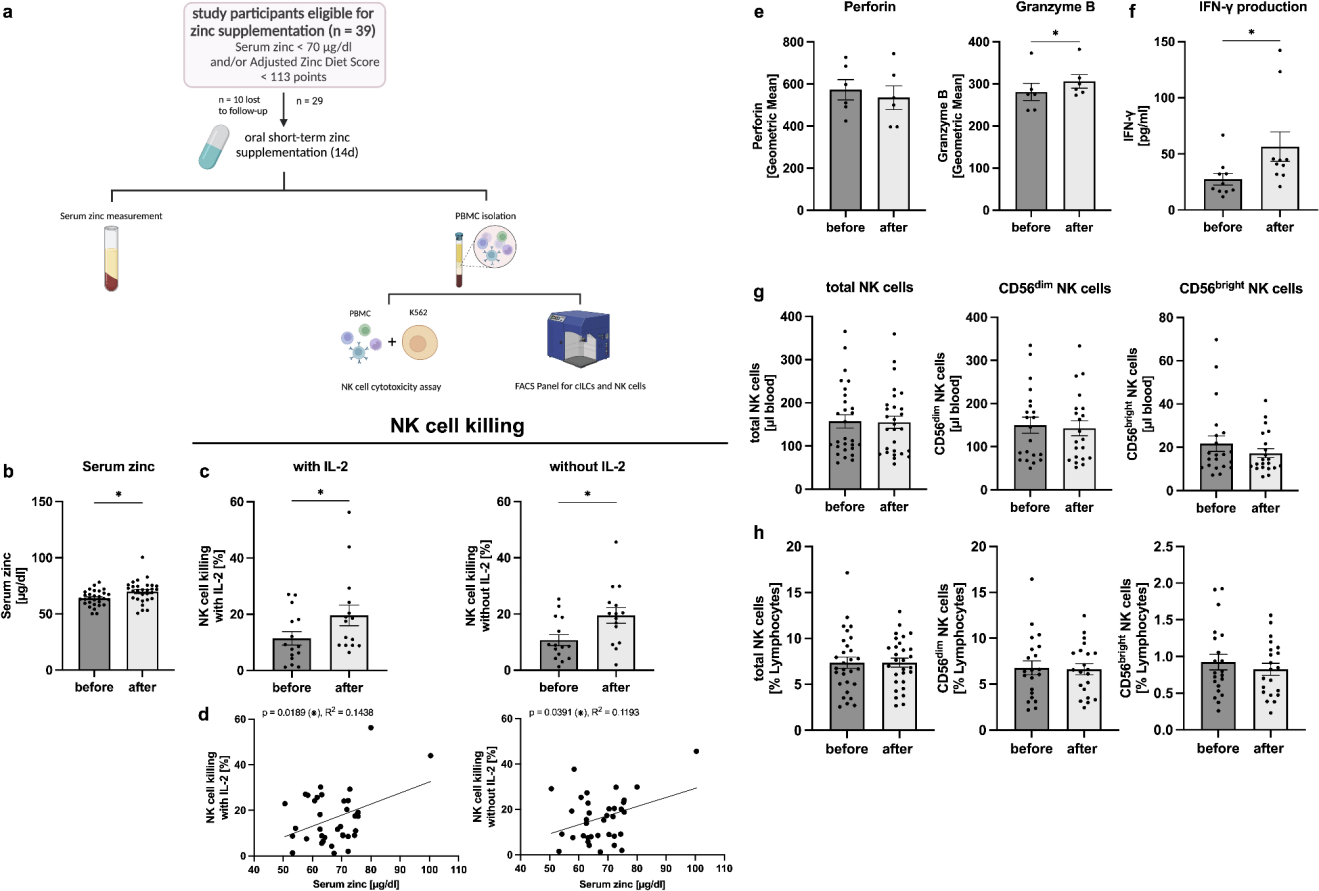
Short-term oral zinc supplementation enhances NK cell functionality. A total of 29 study participants, previously classified as ZD based on serum zinc concentrations and/or FFQ scores, received short-term oral zinc supplementation for 14 days Fig.  1A). After this period, the same experiments were performed. Results before and after zinc supplementation were compared **(A)**. Bar graphs showing serum zinc concentrations (*n* = 29) **(B)**. Bar graphs showing NK cell killing with and without IL-2 stimulation before and after zinc supplementation (*n* = 15) **(C)**. NK cell cytotoxicity of each study participant before and after zinc supplementation is plotted against the corresponding serum zinc concentration (*n* = 36–38, 18–19 pairs) **(D)**. Graph bars show the geometric mean for perforin and granzyme B before and after zinc supplementation (*n* = 6) **(E)**. After PBMCs were co-cultured with K562 cells for 5 h during the NK cell cytotoxicity assay, IFN-γ production was quantified by ELISA shown in bar graphs (*n* = 10) **(F)**. Bar graphs show total NK (*n* = 28–29), CD56^dim^ (*n* = 21), and CD56^bright^ (*n* = 21) NK cell counts **(G)** and frequencies **(H)** before and after zinc supplementation. All data are considered as paired parameters and are presented as the mean ± SEM **(B-C and E-H)**. Statistical significance was calculated by Wilcoxon test in case of non-normal distribution **(C**,** F**,** and G)**, and by paired t-test in case of normal distribution **(B**,** E**,** and H)**. Correlations were determined by the Spearman rank correlation coefficient (Spearman r) **(D)**. * *p*-value < 0.05, ** *p*-value < 0.01, *** *p*-value < 0.001

Our previous findings indicate that neither dietary patterns nor the initial zinc status of the study participants significantly affected cILC counts and frequencies or NK cell counts, frequencies, and cytotoxicity. Two previous in vivo studies focusing on elderly individuals showed that zinc supplementation for one month enhanced NK cell cytotoxicity [34, 35]. Therefore, our next step was to investigate how short-term oral zinc supplementation for 14 days in young and healthy adults eligible for supplementation influences these parameters. Of the 54 study participants, 10 omnivores, 15 vegetarians, and 14 vegans met the criteria for zinc supplementation, as determined by FFQ scores (< 113 points) [26] and/or serum zinc measurements (< 70 µg/dl) [24]. Of these participants, 29 received short-term oral zinc supplementation, while 10 were lost to follow-up. The mean duration of zinc supplementation was 15.10 days ± 4.030 (mean ± SD) (Fig. 1B). After this period, the same experiments were performed (Fig. 4A). To monitor adherence, serum zinc concentrations were measured after zinc supplementation. As expected, zinc supplementation significantly increased serum zinc concentrations in participants from 63.91 µg/dl ± 7.043 (mean ± SD) to 69.61 µg/dl ± 10.02 (Fig. 4B).

Our first objective was to assess NK cell cytotoxicity after 14 days of zinc supplementation and compare it individually with baseline values before supplementation. NK cell cytotoxicity, both with and without IL-2 stimulation, was significantly increased after short-term oral zinc supplementation (Fig. 4C). To further confirm the hypothesis of a zinc-dependent NK cell killing activity, NK cell cytotoxicity of each individual before and after zinc supplementation was plotted against the corresponding serum zinc concentration. Serum zinc levels were significantly positively correlated with NK cell cytotoxicity, both with (R^2^ = 0.1438, *p* = 0.0189) and without (R^2^ = 0.1193, *p* = 0.0391) IL-2 stimulation (Fig. 4D). In conclusion, our results reveal that short-term oral zinc supplementation significantly increases NK cell cytotoxicity in healthy and young adults with a significant positive correlation between NK cell cytotoxicity and serum zinc levels.

### Short-term oral zinc supplementation increases granzyme B levels in NK cells and IFN-γ-production in PBMCs

We next aimed to investigate potential zinc-mediated mechanisms underlying enhanced NK cell cytotoxicity after zinc supplementation. To this end, we stained PBMCs for perforin and granzyme B expression. We observed no significant differences in perforin levels before and after zinc supplementation. However, there was a significant increase in granzyme B post-supplementation (Fig. 4E). This increase in granzyme B levels is consistent with the observed increase in NK cell cytotoxicity in PBMCs following zinc supplementation.

We further quantified the IFN-γ response in the supernatants after 5 h of PBMC co-cultured with K562 cells during the NK cell cytotoxicity assay. As shown in Fig. 4F, IFN-γ levels in the supernatants were significantly higher after zinc supplementation compared to the baseline values.

To verify whether zinc specifically enhances NK cell cytotoxicity by enhancing NK cell functionality, we further compared NK cell counts and frequencies before and after zinc supplementation. Both, cell counts (Fig. 4G) and frequencies of total NK cells, CD56^dim^ and CD56^bright^ NK cells (Fig. 4H) remained stable after zinc supplementation. In conclusion, short-term oral zinc supplementation improves NK cell functionality, possibly through increased granzyme B production and elevated IFN-γ levels, both of which contribute to the enhanced NK cell cytotoxicity. Since NK cell counts and frequencies remained stable after supplementation, these findings suggest that zinc supplementation affects NK cells functionality rather than altering NK cell counts and frequencies.

### Short-term oral zinc supplementation decreases cILC counts and frequencies

**Fig. 5:**
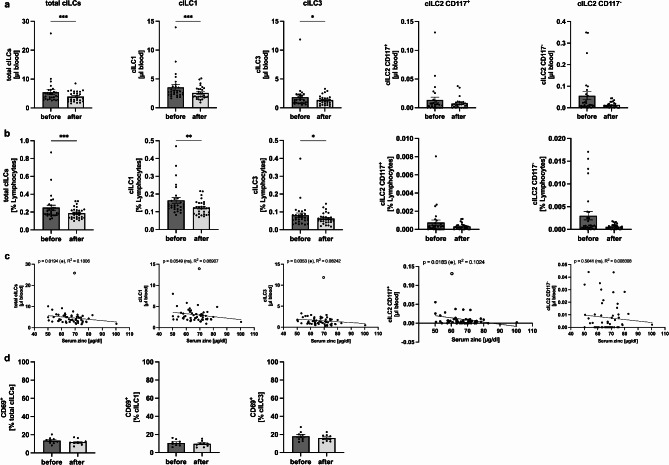
Short-term oral zinc supplementation decreases cILC counts and frequencies. After PBMC isolation by density centrifugation, cILC counts and frequencies were analyzed by flow cytometry and were compared to the baseline value before zinc supplementation. Bar graphs show total cILC, cILC1, cILC2 CD117^+/−^ and cILC3 counts (*n* = 28) **(A)** and frequencies (*n* = 29) **(B)** before and after zinc supplementation. cILC counts of each study participant for all cILC subsets before and after zinc supplementation are plotted against the corresponding serum zinc concentrations (*n* = 56, 28 pairs) **(C)**. Bar graphs showing the percentages of CD69^+^ total ILCs, ILC1s, and ILC3s are compared before and after zinc supplementation (*n* = 9) **(D)**. All data are considered as paired parameters and are presented as the mean ± SEM **(A**,** B**,** and D)**. Statistical significance was calculated by Wilcoxon test in case of non-normal distribution **(A**,** B**,** and D)**, and by paired t-test in in case of normal distribution **(D)**. Correlations were determined by the Spearman rank correlation coefficient (Spearman r). Significances were calculated with one outlier removed. However, the graphs are shown with the outlier **(C)**. * *p*-value < 0.05, ** *p*-value < 0.01, *** *p*-value < 0.001

As the effect of serum zinc levels on cILCs is still unknown, we aimed to investigate the cell counts and frequencies of cILCs following short-term oral zinc supplementation. Both, total cILC counts (Fig. 5A) and frequencies (Fig. 5B) were significantly decreased after zinc supplementation, mainly driven by a decrease in the cILC1 and cILC3 subsets. Although the cILC2 CD117^+/−^ subsets also showed a decreasing trend, this change was not statistically significant. To further support these findings, we plotted cILC counts of each study participant for all cILC subsets before and after zinc supplementation against the corresponding serum zinc concentrations. Our data demonstrate an inverse correlation between serum zinc levels and cILC counts, with significant correlations observed for total cILCs (R^2^ = 0.1006, *p* = 0.0194) as well as the cILC3 (R^2^ = 0.08242, *p* = 0.0353) and cILC2 CD117^+^ (R^2^ = 0.1024, *p* = 0.0183) subsets (Fig. 5C). The cILC1 (R^2^ = 0.06907, *p* = 0.0549) and cILC2 CD117^−^ (R^2^ = 0.008308, *p* = 0.5041) subset showed the same trend, however it was not statistically significant.

As we observed an almost significant trend in CD69^+^ cILCs between ZA and ZD study participants (Fig. 2E), we also evaluated CD69 expression on cILCs before and after zinc supplementation. However, in total cILCs and in the cILC1 and cILC3 subsets, no significant differences in CD69 expression were observed. (Fig. 5D). In summary, these finding show that short-term oral zinc supplementation for two weeks leads to a significantly decrease in absolute cell counts and frequencies of cILCs, mainly driven by a decline of cILC1 and cILC3 subsets.

## Discussion

### Summary

Our study is the first to investigate the effects of dietary habits, correlated serum zinc levels, and short-term oral zinc supplementation on cILC counts and frequencies, as well as NK cell functionality, in young and healthy adults. Our findings indicate that neither dietary patterns nor baseline serum zinc levels influence these parameters. However, short-term oral zinc supplementation for two weeks significantly reduces cILC counts and frequencies while enhancing NK cell cytotoxicity, potentially through elevated granzyme B levels and increased IFN-γ production.

### Zinc’s role in NK cell functionality

In the literature, there is some evidence that NK cells are dependent on zinc. A recent in vitro study from our institute has shown a significant increase in NK cell cytotoxicity in PBMCs from healthy donors due to short (1 h) prior in vitro preincubation with physiological doses of zinc [27]. Similarly, two in vivo studies focusing on elderly individuals have shown that daily zinc supplementation for one month significantly enhances NK cell cytotoxicity [34, 35]. Our findings are consistent with these studies, as we observed a significant increase in NK cell cytotoxicity among zinc-deficient young and healthy volunteers after supplementation with 10 mg of zinc daily for 14 days. In addition, serum zinc levels were found to be positively correlated with NK cell cytotoxicity in zinc-deficient patients independent of the dietary pattern. Of note, most of the study participants supplemented with zinc were female (93%), limiting the interpretability of potential sex-specific differences in immune response to zinc supplementation.

Since zinc is known to affect cellular activity through multiple mechanisms, we investigated potential zinc-dependent cellular pathways underlying increased NK cell cytotoxicity. Given that perforin and granzyme B release are key effector mechanisms of NK cells [5], our data revealed a significant increase in granzyme B expression, but not perforin. In contrast, our previous research showed a significant increase in perforin after short (1 h) in vitro preincubation of PBMCs with 50 µM zinc [27]. These differences in the results may be due to the experimental setups, as our current study involved an in vivo zinc supplementation rather than a short in vitro preincubation. Notably, other in vivo studies in mice have also reported an increase in granzyme B transcription released by cytotoxic T cells upon antigen-stimulation by the addition of zinc [36].

Moreover, several interleukins (IL-2, IL-12, IL-15, and IL-18) [37, 38] and interferons (IFN-γ, IFN-α, IFN-β) [39, 40] are known to stimulate NK cell activity, and many of them have also been described to be highly zinc-dependent. For example, previous studies have shown that zinc supplementation enhances IL-2- [24], IFN-γ- [41] and IFN-α-secretion [42], underscoring the complexity of cellular regulation. Our data also show a significant increase in the IFN-γ production of PBMCs co-cultured with K562 cells for 5 h after zinc supplementation. Since IFN-γ has been demonstrated to enhance NK cell activity [43], it is plausible that the observed increase in IFN-γ production following zinc supplementation—potentially through autocrine and paracrine mechanisms—may contribute to the observed enhancement in NK cell cytotoxicity.

Previous studies have demonstrated that NK cell cytotoxicity in PBMCs increases with an increase in the absolute number of NK cells [44]. To verify whether zinc specifically enhances NK cell cytotoxicity through an enhanced NK cell functionality, we further compared NK cell counts and frequencies before and after zinc supplementation. Our analysis revealed that cell counts as well as frequencies of total NK cells, CD56^dim^, and CD56^bright^ NK cells remained stable after zinc supplementation. This suggests that zinc supplementation primarily affects NK cell functionality rather than their total cell numbers. In conclusion, NK cell functionality appears to be highly zinc-dependent and can be enhanced through short daily zinc supplementation. However, further studies are needed to explore whether increased NK cell functionality is sustained during prolonged or long-term zinc supplementation.

In our study, we did not observe significant differences in NK cell cytotoxicity between omnivores, vegetarians, and vegans, nor between participants classified as ZA or ZD during initial sampling. We hypothesize that in the absence of zinc supplementation, factors other than zinc, such as sex [45], physical exercise [46], and the menstrual cycle [47] may also influence NK cell activity, potentially mitigating the effects of zinc in a steady-state condition. Indeed, NK cell cytotoxicity with and without IL-2 stimulation was higher in males compared to females. Our data demonstrated a significantly lower CD69 expression on both CD56^dim^ and CD56^bright^ NK cells in individuals classified as ZD compared to those classified as ZA. As CD69 is a well-established early activation marker [32], this might suggest that NK cells are less activated in ZD individuals. However, as we observed no differences in NK cell functionality between ZA and ZD study participants, the physiological impact of reduced CD69 expression needs to be further investigated.

### Zinc and cILC counts and frequencies

Innate Lymphoid Cells (ILCs), like NK cells, are key components of the innate immune system. They can be broadly divided into two types: Tissue resident ILCs (tILCs), which closely resemble the function of CD4^+^ T helper cell subsets, and peripheral blood circulating ILCs (cILCs), whose specific functions remain less understood [14]. cILC cell counts and frequencies vary depending on different clinical contexts and comprehensive studies examining the individual quantitative contribution of factors influencing cILC counts and frequencies are still limited [14]. While some research has suggested an association between food uptake and ILC effector functions in the gastrointestinal tract [48, 49], the effects of a specific diet and the resulting trace element levels, such as zinc, on cILC counts, frequencies and composition are still unclear.

Our results indicate that neither dietary patterns nor baseline zinc status significantly affect cell counts, frequencies, or composition of cILCs in the blood. Several studies suggest that ILC counts, frequencies and composition might be established early in life, particularly during pregnancy, the fetal period, and the neonatal phase [50, 51]. Especially ILC3s are strongly influenced by microbial signals derived from maternal microbiota during pregnancy, which are crucial for the establishment of the intestinal ILC3 population [50]. Additionally, a study investigating ILC frequencies in HIV-positive and HIV-negative pediatric cohorts in sub-Saharan Africa demonstrated a persistent depletion of all cILC subsets. Unlike CD4^+^ T cells, these cILCs were not restored by long-term antiretroviral therapy unless initiated at birth [51], further supporting the notion that cILC cell counts, frequencies and composition might be determined early in life. Hence, cILCs might not be strongly influenced by a particular dietary pattern and the resulting zinc status later in life.

However, our findings show a significant reduction in total cILC counts and frequencies following a two-week daily oral zinc supplementation. This decrease was primarily driven by a reduction in the cILC1 and cILC3 subsets. In support of these observations, our data showed an inverse correlation between serum zinc levels and cILC counts, with significant correlations found for total cILCs as well as the cILC3 and cILC2 CD117^+^ subsets. One possible explanation for these results might be an increased activation of cILCs due to zinc supplementation. Prior to zinc supplementation, individuals classified as ZD in serum zinc showed a near significant trend towards reduced CD69 expression on total cILCs as well as on cILC1 and cILC3. Given that CD69 is an early activation marker and an indicator of tissue residency [32], this indicates that cILCs in ZD individuals might be less activated compared to those with adequate zinc levels. This supports the hypothesis that zinc supplementation might enhance cILC activation and might promote their migration into tissues, which possibly accounts for the reduced cILC counts and frequencies in peripheral blood post-supplementation. No change in activation status, as measured by CD69 expression, was observed after supplementation, likely because activated cILCs might have migrated to tissues, rendering them undetectable in the circulation.

Depletion of cILCs from peripheral blood has been observed in various clinical conditions, for example in COVID-19 patients. The remaining cILCs exhibited a more activated profile and dysregulated receptor expression compared to those of healthy controls, suggesting enhanced activation and differential recruitment to tissues, potentially contributing to antiviral defense [52]. Furthermore, age-related changes also influence cILC composition, in particular a significant decrease in cILC1 cell counts from infancy to adolescence [31], suggesting that cILCs might play a role in maintaining tissue ILC pools during adulthood, as suggested by other studies [14, 53]. Based on these findings, we hypothesize that zinc supplementation most likely might promote a pro-migratory profile in cILCs, therefore leading to the observed decrease in cILC counts and frequencies in peripheral blood.

Of note, further research is needed to investigate the mechanisms involved in the zinc-induced decrease in cILC counts and frequencies, such as analysis of homing molecules or receptors. Moreover, it would be valuable to analyze the decrease in cILCs in a time-dependent manner, determining when their decline begins or when they migrate into tissues. Understanding whether cILCs are restored in the circulation during prolonged zinc supplementation and whether their levels are being replenished once supplementation is discontinued would also provide important insights. Of note, in our experimental setup, we observed that the number of cILC2s detected by flow cytometry was lower compared to similar studies [54]. For instance, a recent study investigating cILC counts and frequencies in healthy adults aged 18–55 years reported that cILC2 accounted for approximately 0.04% of lymphocytes [54]. In contrast, cILC2 frequency in our study was only about 0.0035% of total lymphocytes. This discrepancy may be partly attributable to differences in age ranges between study populations. However, the precise cause remains unclear and needs to be further investigated.

### Possible health implications of zinc deficiency and routine zinc supplementation in young and healthy adults

In our study cohort, 72% of all participants were classified as ZD based on FFQ scores and/or serum zinc concentrations, making them suitable candidates for zinc supplementation. Surprisingly, even in the omnivore group, which was originally intended to serve as a control group with presumed high serum zinc concentrations, the mean serum zinc level (71.33 µg/dl ± 7.818 (mean ± SD)) was remarkably low compared to previous studies [24]. Nearly half (48%) of the omnivores were classified as ZD, challenging the assumption that all omnivores would have sufficient zinc levels. The relatively low serum zinc concentrations observed in our study may be attributed to the increasing prevalence of “flexitarian” diets, especially among young people, in which meat consumption is reduced despite following an omnivorous diet [55]. Another contributing factor could be the timing of the blood draws, which were conducted in the afternoon. By this time, fluid uptake throughout the day may have caused a slight dilution of serum zinc levels. The predominance of females especially in the vegetarian and vegan cohorts, who are more likely to adopt plant-based diets [56], may also have influenced overall serum zinc levels. According to FFQ results, women often consumed smaller quantities of food, leading to lower FFQ scores, which may partially explain the lower serum zinc levels across all groups.

Recent studies have reported a notable increase in early-onset cancers and inflammatory bowel diseases (IBD) in the younger population [57–59], raising public health concerns. Given the important role of zinc in immune function and tissue homeostasis, and thus in the prevention of such diseases [11, 60–62] our findings are especially relevant as 72% of our study cohort was considered ZD by the means of the FFQ and/or serum zinc measurements independent of their diet. Furthermore, zinc deficiency in IBD patients has been associated with adverse disease-specific outcomes [63]. Here, we demonstrated that zinc supplementation in ZD participants significantly improved NK cell functionality, as evidenced by increased NK cell cytotoxicity, granzyme B levels, and IFN-γ production. The improvement of these parameters due to zinc supplementation supports the benefits of daily zinc supplementation, as proper NK cell function is crucial for viral defense, tumor suppression, and overall immune equilibrium [4].

In addition, we observed a significant decrease in cILC counts and frequencies following zinc supplementation. We hypothesize, that the observed reduction might be most likely due to a possible migration of cILCs into tissues, where they play a pivotal role in regulating immune responses, tissue homeostasis, and inflammation [11]. In particular, cILC3s, which are thought to be progenitors to all cILC subsets and NK cells [14], might be essential in this context to replenish tissue resident ILCs, thereby improving barrier functionality. All these processes might be especially important in the context of preventing IBD and promoting gut homeostasis [64, 65]. However, it is important to recognize that immune surveillance, particularly tumor control, involves far more complex regulatory mechanisms than NK cell activity alone. Other immune cells, such as regulatory T cells (Treg) and T helper cells (Th) also play important roles [66, 67], yet zinc has been shown to influence these cell types as well [68–70]. However, further research is needed to fully understand the role of zinc in these processes and its potential in disease prevention.

## Conclusions

Our study is the first to investigate the effects of dietary habits, correlated serum zinc levels, and short-term oral zinc supplementation on cILC counts and frequencies, as well as NK cell functionality, in young and healthy adults. Our findings indicate that neither dietary patterns nor baseline serum zinc levels influence these parameters. However, short-term oral zinc supplementation for two weeks significantly reduces cILC counts and frequencies while enhancing NK cell cytotoxicity, potentially through elevated granzyme B levels and increased IFN-γ production. Overall, when our findings are considered in the context of the existing literature, it becomes evident that adequate zinc uptake is crucial not only for individuals who follow a plant-based diet, such as vegetarians and vegans, but also for omnivores who reduce meat consumption. Considering the growing trend towards flexitarian diets [55], ensuring sufficient zinc uptake is essential for maintaining proper immune function.

Thus, routine zinc supplementation may offer a simple yet effective strategy for enhancing immune defense and potentially promoting disease prevention across different dietary groups.

## Electronic supplementary material

Below is the link to the electronic supplementary material.

**No significant sex-specific differences in total NK cell and cILC counts, frequencies, or NK cell cytotoxicity**PBMCs were isolated by density gradient from peripheral blood of young and healthy adults. Total NK cell and total cILC counts and frequencies were analyzed by flow cytometry, and NK cell cytotoxicity assay was performed. Bar graphs show total cILC counts and frequencies **(A), **total NK cell counts and frequencies **(B)**, and percentages of PI-positive K562 cells, quantifying NK cell killing **(C)**, between male (n = 12-13) and female (n = 31-41) participants. All data are considered as unpaired parameters and are presented as the mean ± SEM **(A-C)**. Statistical significance was calculated by Mann-Whitney test **(A-C)**. * p-value < 0.05, ** p-value < 0.01, *** p-value < 0.001

Supplementary Material 2

Supplementary Material 3

## Data Availability

The datasets used and/or analyzed during the current study are available from the corresponding author on reasonable request.

## Abbreviations

AAS: Atomic absorption spectroscopy
AZDS: Adjusted zinc diet score
CFDA-SE: CFDA-SE: Carboxyfluorescein diacetate succinimidyl ester
cILCs: Circulating innate lymphoid cells
CRTH: Prostaglandin DP2 receptor
FasL: Fas ligand
FCS: Fetal calf serum
FFQ: Food frequency questionnaire
HLA: Human leucocyte antigen
IBD: Inflammatory bowel diseases
IFN: Interferon
IL: Interleukin
ILCs: Innate lymphoid cells
KIR: Killer cell immunoglobulin-like receptor
NK cell: Natural killer cell
OM: Omnivore
PBMC: Peripheral blood mononuclear cells
PI: Propidium iodide
SD: Standard deviation
SEM: Standard error of the mean
tILCs: Tissue resident innate lymphoid cells
TRAIL: Tumor necrosis factor-related apoptosis-inducing ligand
VG: Vegan
VT: Vegetarian
VIP: Vasoactive intestinal peptide
ZA: Zinc adequate
ZD: Zinc deficient
